# Cerebellar contributions to fear-based emotional processing: relevance to understanding the neural circuits involved in autism

**DOI:** 10.3389/fnsys.2023.1229627

**Published:** 2023-11-21

**Authors:** Sabina Couto-Ovejero, Jingjing Ye, Peter C. Kind, Sally M. Till, Thomas C. Watson

**Affiliations:** Simons Initiative for the Developing Brain, Centre for Discovery Brain Sciences, University of Edinburgh, Edinburgh, United Kingdom

**Keywords:** cerebellum, fear, autism, prediction error, network

## Abstract

Cerebellar networks have traditionally been linked to sensorimotor control. However, a large body of evidence suggests that cerebellar functions extend to non-motor realms, such as fear-based emotional processing and that these functions are supported by interactions with a wide range of brain structures. Research related to the cerebellar contributions to emotional processing has focussed primarily on the use of well-constrained conditioning paradigms in both human and non-human subjects. From these studies, cerebellar circuits appear to be critically involved in both conditioned and unconditioned responses to threatening stimuli in addition to encoding and storage of fear memory. It has been hypothesised that the computational mechanism underlying this contribution may involve internal models, where errors between actual and expected outcomes are computed within the circuitry of the cerebellum. From a clinical perspective, cerebellar abnormalities have been consistently linked to neurodevelopmental disorders, including autism. Importantly, atypical adaptive behaviour and heightened anxiety are also common amongst autistic individuals. In this review, we provide an overview of the current anatomical, physiological and theoretical understanding of cerebellar contributions to fear-based emotional processing to foster further insights into the neural circuitry underlying emotional dysregulation observed in people with autism.

## Introduction

For decades the cerebellum has been ascribed functions related exclusively to motor control and motor learning. Many of the major theories of cerebellar information processing within this field have been based around the concept of internal models, which are neural representations facilitating comparison of predicted and actual motor states, allowing for efficient behavioural performance adaptation driven by prediction error (PE; Wolpert et al., 1998; Popa and Ebner, 2018). Within the motor domain, sensorimotor relationships are stored in an updated model within the central nervous system, in which the cerebellum plays a critical role. Sensory feedback that diverges from predictions is encoded as PE, which is then used to refine the internal model. Theoretical and physiological studies have highlighted the important role of the inferior olive, a pre-cerebellar nuclei, in providing a ‘teaching signal’ via climbing fibre projections to the Purkinje cells in the cerebellar cortex. This teaching or error signal is thought to be vital for modulating predictive states in the cerebellum (see Apps and Garwicz, 2005; Ramnani, 2006; Lang et al., 2017 for reviews on this topic). Recent studies have revealed that cerebellar functions also extend to cognitive and fear-based emotional processes (Strick et al., 2009; Koziol et al., 2014; Apps and Strata, 2015; Strata, 2015; Van Overwalle et al., 2020a). The question of how the cerebellum contributes to such non-motor information processing has received much attention over the last two decades (Strick et al., 2009; Koziol et al., 2014; Van Overwalle et al., 2020a). One of the most prominent suggestions is that the cerebellar internal model system may process information uniformly (Popa and Ebner, 2018), and that computations are determined by closed-loop anatomical connectivity of circumscribed cerebellar regions to either motor or non-motor brain regions, respectively (Ramnani, 2006). Thus, it is plausible that cerebellar contributions to fear-based emotional processing may be centred around computation of predictive models as is thought to occur in the motor domain (see Ciapponi et al., 2023 for computational perspective). These models may be updated by PE and supported through reciprocal anatomical, and functional connectivity with various structural hubs across the forebrain, midbrain and limbic circuits (Ernst et al., 2019; Frontera et al., 2020).

Autism is an umbrella term covering a group of complex neurodevelopmental conditions that become evident in childhood and can co-occur with intellectual disability and epilepsy. Diagnostic criteria are based on classic traits such as atypical social interaction and reciprocal communication as well as repetitive and restricted interests (American Psychiatric Association, 2013). Emotional dysregulation is also commonly associated with autism (Mazefsky et al., 2013; Samson et al., 2015; Macari et al., 2018; Conner et al., 2020). This altered capacity to modulate arousal and emotional responses to support adaptive and typical social behaviour may be linked to other features that frequently co-occur in autistic individuals including heightened anxiety, difficulty adapting to change and altered stimulus–response associations (Burke and Cerniglia, 1990; Green et al., 2013; Davis Iii et al., 2014; Kerns and Kendall, 2014; Conner et al., 2020).

Although our understanding of the neurobiological mechanisms leading to autism symptoms remains incomplete, it has been proposed that differences in predictive encoding may play a critical role (Kelly et al., 2021; Stoodley and Tsai, 2021). Predictive coding is a type of Bayesian inference the brain uses to efficiently deal with enormous sensory information (Friston, 2005). In addition to receiving sensory input, the brain also actively predicts incoming stimuli (de-Wit et al., 2010); using the discrepancies between expectation and sensory input, PE, the brain continually generates and updates its perception of the external world (Lawson et al., 2014). The precision of predictive coding is subserved by the post-synaptic excitability of cells encoding PEs, which is influenced by neuromodulator systems commonly affected in autism (Lawson et al., 2014). Autistic individuals show aberrant precision of encoding (Friston et al., 2013; Lawson et al., 2014) and may be biased towards a rote memorization learning style (Qian and Lipkin, 2011). Furthermore, studies have revealed atypical anatomical and functional cerebello-cerebral connectivity in autistic individuals (Hanaie et al., 2013; Stoodley et al., 2017; Arnold Anteraper et al., 2019; Kelly et al., 2020; Gaudfernau et al., 2022) and corresponding mouse models (Tsai, 2016; Stoodley et al., 2017; Badura et al., 2018; Kelly et al., 2020), which may underlie widespread changes in predictive encoding. However, there is limited research on the processing of PEs in cerebellar circuits during fear-based emotional processing in autism or neurodevelopmental disorders more generally.

Thus, in this review, we will describe known cerebellar roles in fear, one of the most well studied emotional domains, in both animal models and humans. We will then describe studies on the intra-cerebellar mechanisms that may underlie such contributions. In our final section, we will attempt to bring together these two areas of research to identify and discuss potential cerebellar contributions to the dysregulation of fear-based emotional processing observed in autistic individuals.

## Fear and the cerebellum

The detection of threat drives the emotional and defensive response of fear. The neural basis of fear has been studied extensively in both humans and animal models for many years (Ledoux, 1998; Apps and Strata, 2015; Onat and Buchel, 2015; Tovote et al., 2015; Garcia, 2017). However, the suggestion that the cerebellum may be involved in fear processing is relatively recent, with the first studies directly addressing this question arising in the early 90s (Supple and Leaton, 1990). Since then, there has been a range of research investigating cerebellar involvement in fear at cellular, circuits and behavioural levels (see Apps and Strata, 2015; Strata, 2015; Hwang et al., 2022 for review). The purpose of this review is not to provide an exhaustive overview of all these studies but rather we will focus on the recently proposed theory that prediction error encoding supports cerebellar contributions to fear and that its aberrance may contribute to the fear-based emotional dysregulation observed in autism.

## Cerebellar gross anatomy

**Figure 1:**
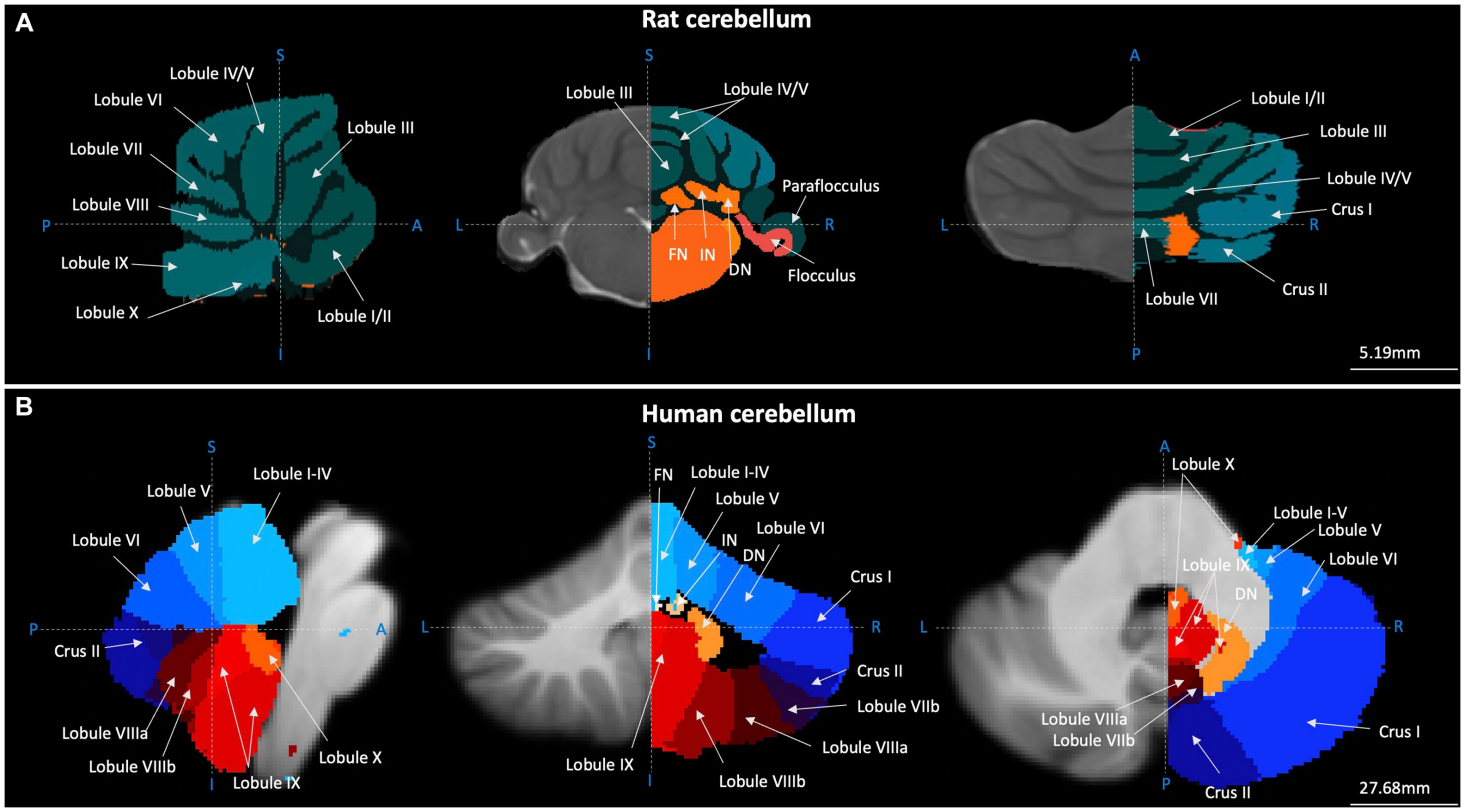
Gross anatomical structure of the cerebellum in rats and humans. Cerebellar topography as visualised using structural MRI data (grey) and cerebellar segmentation (coloured) using **(A)** the Fisher 344 Rat Brain atlas (Goerzen et al., 2020) and **(B)** the human Probabilistic Cerebellar Atlas (Diedrichsen et al., 2009). Sagittal, coronal, and horizontal planes depicted in left, centre and right panels, respectively. Blue dashed lines and letters indicate orientation. A-P, anterior–posterior; I-S, inferior–superior; L-R, left–right; FN, fastigial nucleus; IN, nucleus interpositus; DN, dentate nucleus. Images were generated using FMRIB Software Library (Jenkinson et al., 2012).

The cerebellum is composed of an outer, folded cortex and deep cerebellar nuclei, which provide output to the rest of the brain (see Figure 1). The cerebellar cortex can be separated into longitudinal regions in the rostro-caudal plane: the vermis (medial), paravermis (intermediate) and hemispheres (lateral; Figure 1). Each of these regions is composed of lobules, or folds in the cerebellar structure [for comprehensive description of cerebellar lobule nomenclature see Apps and Hawkes (2009)]. The compartmental organisation of the cerebellum provides the structural basis for the theory of lobule-specific involvement in motor/cognitive function (Stoodley and Schmahmann, 2010; Stoodley et al., 2012). Classically, cerebellar cortical architecture is thought to be homogenous across lobules and as such, computations are considered uniform across regions (see Stoodley and Schmahmann, 2010 for discussion of the universal cerebellar transform). Thus, cerebellar information processing is thought to be highly dictated by its input from and output to other regions of the brain. Recent studies have questioned the degree of physiological uniformity in cerebellar circuits (Cerminara et al., 2015) and functional units (modules) within the cerebellum may have overlapping roles in behaviour (Cerminara and Apps, 2011). However, findings on cerebellar physiological heterogeneity are primarily based on studies of the motor system and it remains an open question as to whether they generalise or extend to processing of non-motor functions, such as emotional control.

For the cerebellum to efficiently contribute to processes such as fear-based emotion, direct anatomical projections to fear-related networks in the brain are required. Therefore, in the following subsection we will provide an overview of known monosynaptic, anatomical connections between cerebellum and fear ‘hubs’ across the brain in both humans and animal models.

## Cerebellar anatomical connections with the fear circuitry

**Figure 2:**
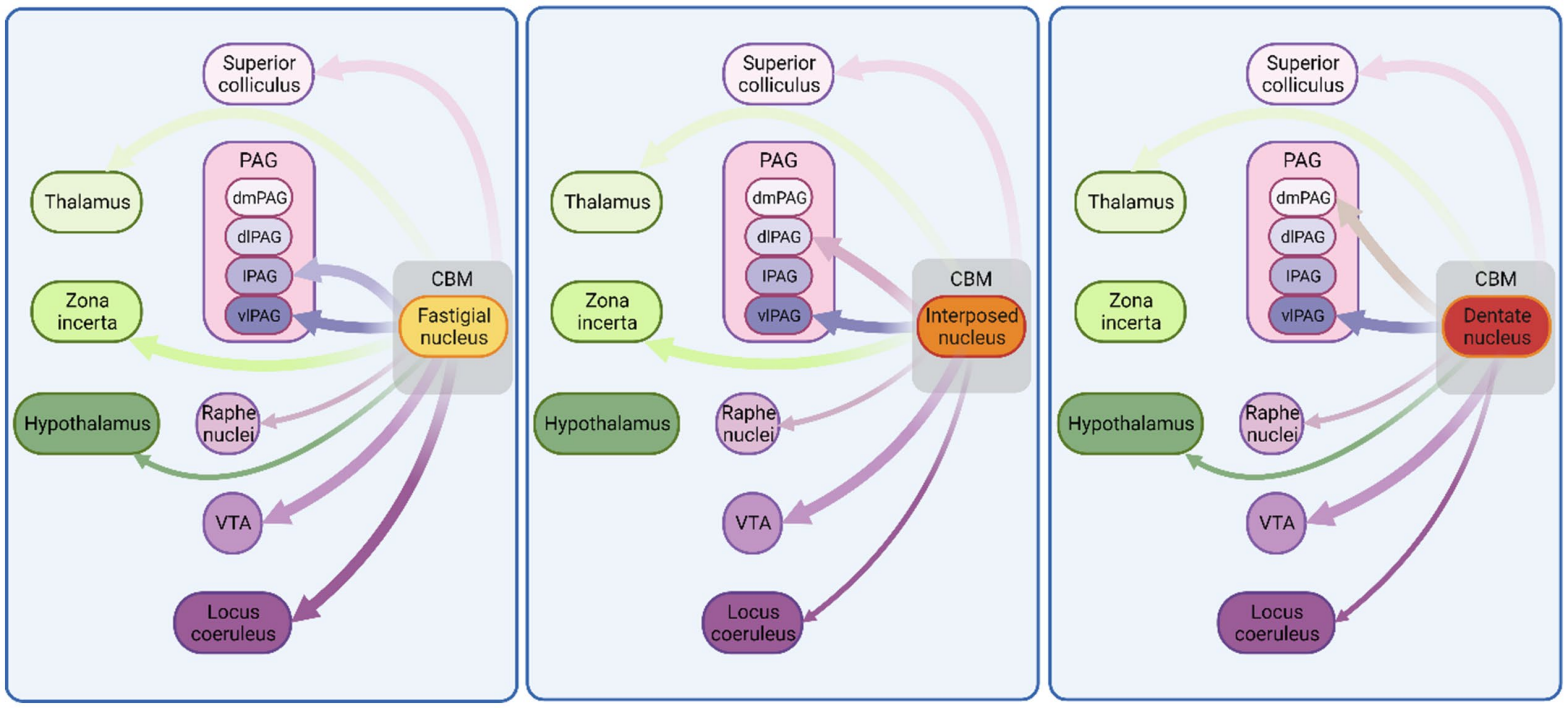
Monosynaptic connectivity between cerebellum and other fear-related brain regions in non-human species. Schematic illustration of connections between cerebellum and known fear-related structures. Exclusively, anatomical mapping data were used to construct this figure. Thickness of arrows indicates projection density. CBM, cerebellum; VTA, ventral tegmental area; PAG, periaqueductal grey; dmPAG, dorsomedial periaqueductal grey; dPAG, dorsal periaqueductal grey; lPAG, lateral periaqueductal grey; vlPAG, ventrolateral periaqueductal grey. See Supplementary Table 1 for list of references used to compile this figure. Figure made with Biorender.com.

**Figure 3:**
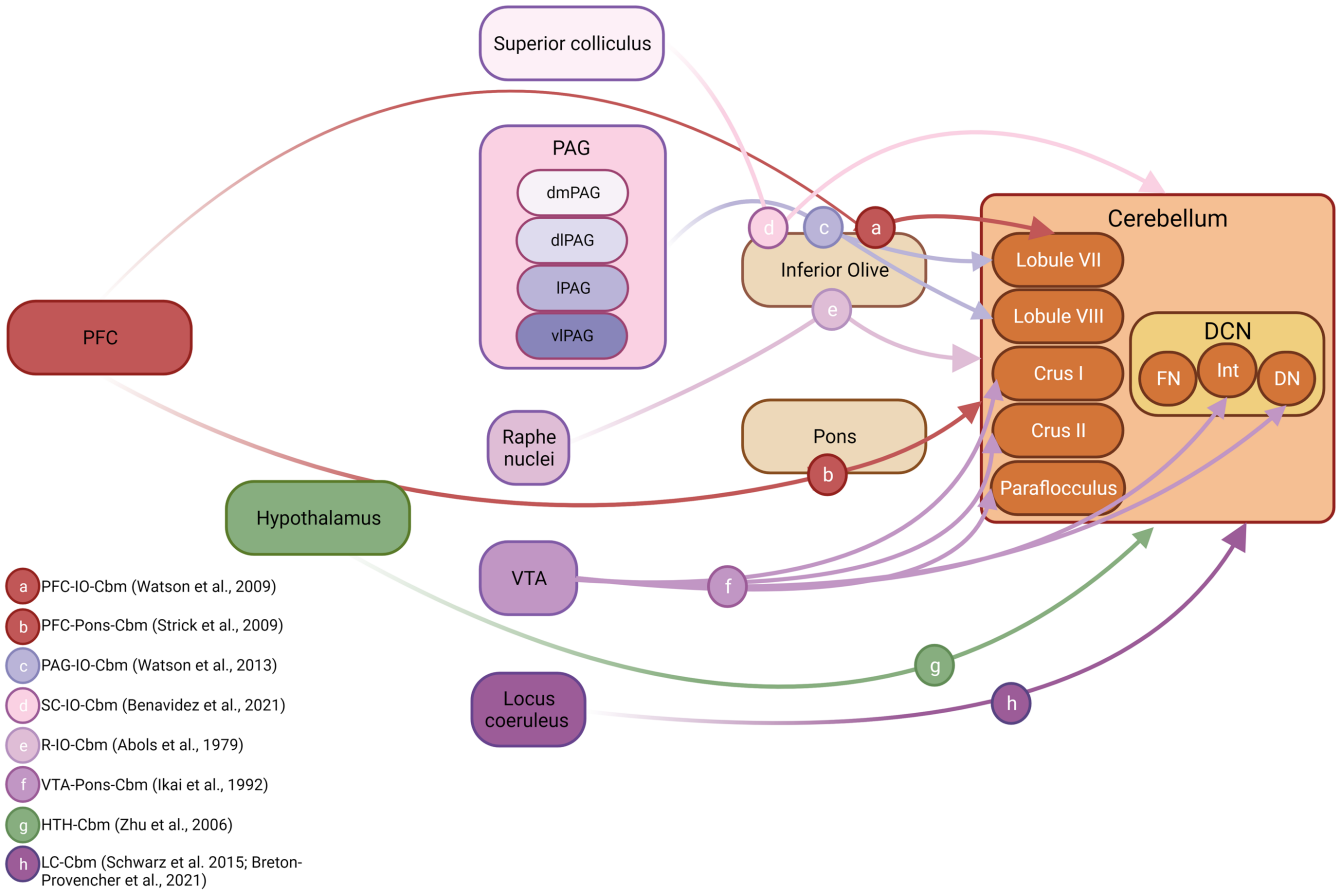
Cerebellar inputs arising from fear-related brain structures in non-human species. Schematic diagram illustrating connections between known fear-related structures and the cerebellum. Both anatomical and physiological mapping data were used to construct this figure. DCN, deep cerebellar nuclei; FN, fastigial nucleus; Int, interpositus nucleus; DN, dentate nucleus; PFC, prefrontal cortex; PAG, periaqueductal grey; dmPAG, dorsomedial periaqueductal grey; dPAG, dorsal periaqueductal grey; lPAG, lateral periaqueductal grey; vlPAG, ventrolateral periaqueductal grey. Inset, references supporting depicted anatomical connections, letter coded. Figure made with Biorender.com. IO, inferior olive cbm, cerebellum SC, superior colliculus R, Raphe nuclei VTA, ventral tegmental area HTH, hypothalamus LC, locus coeruleus.

**Figure 4:**
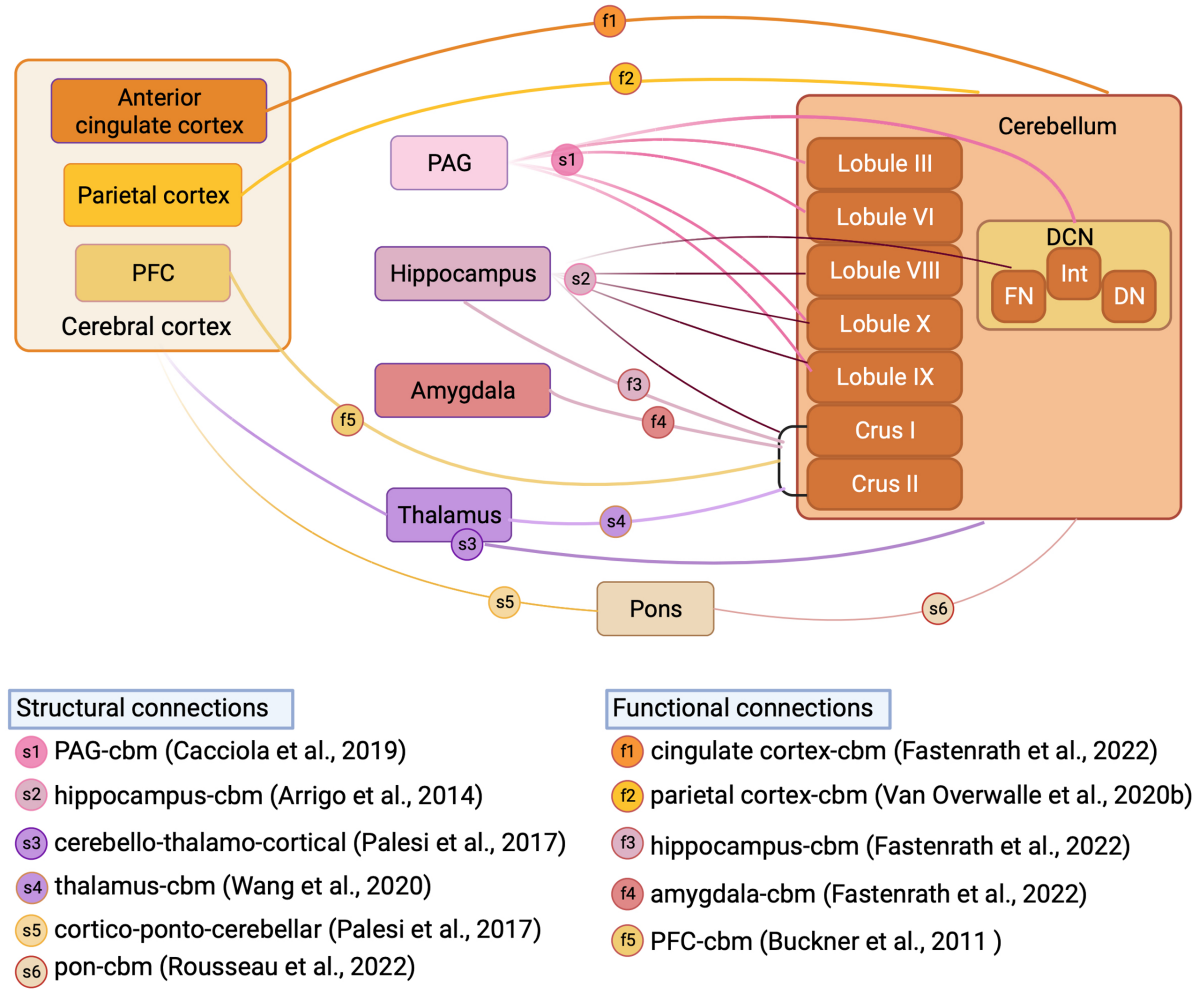
Structural and functional cerebellar connections with fear-related regions reported in human studies. Schematic illustration of structural connections (reported from structural MRI studies) and functional connections (reported from fMRI studies) between cerebellum and fear-related regions in Human. DCN, deep cerebellar nuclei; FN, fastigial nucleus; Int, interpositus nucleus; DN, dentate nucleus; PFC, prefrontal cortex; PAG, periaqueductal grey; Inset, references supporting structural and functional connections, letter coded. Figure made with Biorender.com.

Cerebellar internal models are thought to fundamentally rely on closed-loop anatomical connectivity with other brain regions. For example, within the motor domain, regions of the cerebellum that receive input from the motor cortex also project back to the same area. This allows for models to constantly receive descending motor commands, update and transmit back to the cerebral cortex (Ramnani, 2006). It has been suggested that an analogous system may support cerebellar contributions to non-motor functions, such as emotion, via connections with brain regions out with the motor realm (Ramnani, 2006; Apps and Strata, 2015; Strata, 2015; Hwang et al., 2022). A detailed anatomical substrate to support this hypothesis is essential to further understanding of the cerebellum and emotion, and in particular, fear. Below, we outline the mono/di-synaptic efferent and afferent anatomical connectivity via which the cerebellum may send (via the cerebellar nuclei) and receive (via the pre-cerebellar nuclei and cerebellar cortex) information from the wider fear network in animal models (see Figures 2, 3) and humans (Figure 4).

## Animal studies: cerebellar monosynaptic connections to the mesencephalic fear regions

The cerebellum projects to multiple regions of the mesencephalon. Here we describe known monosynaptic connectivity, as identified by neuroanatomical tracing in animal models, with those regions of the mesencephalon that are often considered as part of the fear network. These data are summarised schematically in Figure 2.

### Cerebellar projections to ventral tegmental area

Ventral tegmental area (VTA) plays important roles in reward, aversion and stress, and as such can be considered a key component of the fear network (Lammel et al., 2012; Bouarab et al., 2019). Of particular relevance to this review, the VTA is critical for the generation of reward PE signals (Hollerman and Schultz, 1998; Schultz, 1998; Schultz, 2016) and dopaminergic neural activity patterns in the VTA have also been linked to modulation of fear extinction learning in animal models (Salinas-Hernandez et al., 2018; Cai et al., 2020). The VTA receives excitatory input from all three cerebellar nuclei (Perciavalle et al., 1989; Carta et al., 2019; Fujita et al., 2020; Judd et al., 2021; Baek et al., 2022). Importantly, recent work in mice has shown that modulation of cerebellar projection to the VTA can drive reward social and stress related behaviours (Carta et al., 2019; Baek et al., 2022).

### Cerebellar projections to superior colliculus

The superior colliculus (SC) has been shown to play important roles in visual fear conditioning (Shang et al., 2018) and threat assessment, in particular to looming stimuli (Evans et al., 2018). It projects heavily to the periaqueductal grey, a key region involved in executing appropriate behavioural responses during threatening situations (Mobbs et al., 2007; Wang et al., 2022). Several authors report glutamatergic projections from the interpositus and fastigial nucleus to all layers of the SC (Gonzalo-Ruiz and Leichnetz, 1987; Gonzalo-Ruiz et al., 1988; Katoh et al., 2000; Fujita et al., 2020; Heiney et al., 2021; Judd et al., 2021) and anatomical studies in rats and cats have shown that all three cerebellar nuclei project to the SC (Hirai et al., 1982; Gonzalo-Ruiz and Leichnetz, 1987). However, an anatomical study in primates highlighted the absence of dentate nucleus projections to SC in this species (Gonzalo-Ruiz et al., 1988).

### Cerebellar projections to periaqueductal grey

The midbrain periaqueductal grey is involved in a variety of diverse functions ranging from sleep-state control (Weber and Dan, 2016; Zhong et al., 2019) to descending modulation of painful stimuli, in addition to well characterised roles in survival behaviours and defensive responses (Mobbs et al., 2007; Wang et al., 2022). The PAG can be anatomically subdivided in to at least four main subdivisions: dorsomedial, dorsolateral, lateral, and ventrolateral (Bandler and Shipley, 1994), which receive excitatory monosynaptic projections from the cerebellar nuclei (Beitz, 1982; Beitz, 1989; Judd et al., 2021). However, the ventrolateral PAG is the only subregion to receive input from all three cerebellar nuclei (Beitz, 1982; Frontera et al., 2020; Fujita et al., 2020; Vaaga et al., 2020; Judd et al., 2021). In contrast, the dorsolateral PAG receives input solely from the interposed nucleus (Gonzalo-Ruiz and Leichnetz, 1990), the dorsomedial PAG solely from the dentate nucleus and the lateral PAG solely from the fastigial nucleus, respectively (Kawamura et al., 1982; Gonzalo-Ruiz et al., 1990; Apps and Strata, 2015).

### Cerebellar projections to locus coeruleus

Locus coeruleus provides the main source of the neuromodulator noradrenaline to the brain. Via its wide neuromodulatory reach, locus coeruleus influences several functions relating to fear, including conditioning and extinction (see Giustino and Maren, 2018 for review). Situated anatomically in close proximity to the cerebellum, it receives input from all three cerebellar nuclei. However, the most prominent cerebellar inputs to locus coeruleus emanate from the fastigial nucleus (Cedarbaum and Aghajanian, 1978; Clavier, 1979; Mezey et al., 1985; Schwarz et al., 2015; Fujita et al., 2020; Kebschull et al., 2020).

### Cerebellar projections to raphe nuclei

As part of the medullary reticular formation, the raphe nuclei are composed primarily of serotonergic neurons and can be subdivided into obscurus, magnus and pallidus nuclei. Generally, the raphe nuclei are involved in functions relating to stress and reward processing (Crawford et al., 2013; Nakamura, 2013; Luo et al., 2015), and in relation to fear, the dorsal raphe in particular has been shown to be critical for extinction learning in rodents (Berg et al., 2014). The obscurus and magnus nuclei receive excitatory projections from all cerebellar nuclei (Asanuma et al., 1983; Langer and Kaneko, 1984; Mezey et al., 1985; Marcinkiewicz et al., 1989; Gonzalo-Ruiz and Leichnetz, 1990). To the best of our knowledge, cerebellar nuclei projections to the pallidus nuclei are yet to be described.

## Cerebellar monosynaptic connections to the diencephalic fear regions

The cerebellum projects to multiple diencephalic regions. Here we describe known monosynaptic connectivity, as identified by neuroanatomical tracing in animal models, with those regions of the diencephalon that may be considered as part of the classical fear network.

### Cerebellar projections to hypothalamus

The hypothalamus has a broad range of functions, all linked to emotional and affective behaviours (Baroncini et al., 2012). Recently, optogenetic experiments in mice have highlighted multiple roles for ventromedial hypothalamic neurons in emotional processes, including fear learning/conditioning (Kunwar et al., 2015) and melanin-concentrating hormone expressing neurons within the lateral hypothalamus have been critically linked to fear extinction learning in mice (Concetti et al., 2020). There is evidence of projections from all three cerebellar nuclei to the lateral and posterior hypothalamus in monkey and tree shrew, respectively (Haines and Dietrichs, 1984; Haines et al., 1990; Apps and Strata, 2015), and also to the dorsomedial hypothalamus in rat (Cavdar et al., 2001). In rats, ventromedial hypothalamus receives inhibitory and excitatory input from the fastigial nucleus (Li et al., 2017).

### Cerebellar projections to thalamus

Thalamus is the main conduit via which cerebellar output is routed to the cerebral cortex (Schafer and Hoebeek, 2018; Habas et al., 2019). It is divided in four regions: anterior, posterior, medial and lateral (Lambert et al., 2017). All thalamic nuclei receive monosynaptic input from the cerebellum, except for the anterior region (Haroian et al., 1981). Whilst there are a vast number of studies charting cerebello-thalamic connectivity in a range of species (see Habas et al., 2019 for review), in relation to emotional processing, the mediodorsal thalamus (MD) has been shown to be particularly involved in both acquisition and extinction of fear memories (Li et al., 2004; Lee et al., 2011) via its connectivity with fastigial nucleus (Frontera et al., 2023). Indeed, several authors reported excitatory projections from the fastigial nucleus and interposed nucleus to the mediodorsal thalamus in mouse (Fujita et al., 2020; Judd et al., 2021; Frontera et al., 2023). Further, studies conducted in monkeys, have identified MD thalamic projections from the interposed and dentate nucleus (Gonzalo-Ruiz and Leichnetz, 1990; Sakai et al., 1996) and from all three cerebellar nuclei in dog (Person et al., 1986; Stepniewska and Kosmal, 1986; Sakai and Patton, 1993).

### Cerebellar projections to zona incerta

Zona incerta supports a wide variety of processes related to visceral function, arousal, locomotion, and nociception (Spencer et al., 1988; Nicolelis et al., 1995) as well as fear modulation (Chou et al., 2018; Venkataraman et al., 2019; Li et al., 2021). It has been shown that stimulation of GABAergic neurons in zona incerta enhances extinction of fear memories (Zhou et al., 2018; Venkataraman et al., 2021). All three cerebellar nuclei send projections to the zona incerta (Kuramoto et al., 2011; Kebschull et al., 2020) with evidence of excitatory connections from the fastigial (Fujita et al., 2020), interpositus and dentate nucleus (Roger and Cadusseau, 1985; Aumann and Horne, 1996; Mitrofanis and deFonseka, 2001) in rat, fastigial and interposed nucleus in cat (Sugimoto et al., 1981), and from interposed nucleus in monkey (Gonzalo-Ruiz and Leichnetz, 1990), respectively.

### Fear network projections to the cerebellum

The cerebellum also receives input from a wide variety of fear related brain structures (see Apps and Strata, 2015 for overview). However, given that the majority of cerebellar input is routed via disynaptic pathways through the precerebellar nuclei (e.g., pons or inferior olive) our understanding of these projections based on strictly anatomical tracing studies is limited. Therefore, below we outline a combination of electrophysiological mapping and, where available, anatomical tracing data describing fear network input to the cerebellum. These data are summarised schematically in Figure 3.

Cerebral cortical inputs from regions such as the prefrontal cortex, that are critical to fear learning, have been described to the cerebellum in both non-human primates (Kelly and Strick, 2003) and rodent (Watson et al., 2009; Suzuki et al., 2012). The pioneering work of Strick and colleagues (see Strick et al., 2009 for overview) first used trans-synaptic viral tracing techniques to reveal dorsolateral prefrontal cortex-ponto-cerebellar projections, restricted heavily to hemispheric Crus I/II and also to vermal lobule VII in primates (Kelly and Strick, 2003). Watson et al. (2009) partially replicated these findings in rodent using electrophysiological mapping techniques to chart the cerebellar regions most heavily activated following electrical stimulation of the prelimbic region. Whilst this study could not categorically identify the anatomical route via which prelimbic inputs reach the cerebellum, based upon the physiological characteristics of the evoked electrophysiological response, it is highly likely that a prelimbic-olivo-cerebellar pathway was activated, and this input was restricted to vermal lobule VII. Using a similar method, Watson et al. (2013) also described PAG-olivo-cerebellar pathways in anaesthetised rats that were topographically restricted to lobule VIII. Anatomical tracing studies have described olivary projections emanating from superior colliculus and raphe nucleus in rodents (Abols and Basbaum, 1979; Benavidez et al., 2021); however, it is unclear which regions of the cerebellum are targeted by these inputs (see Figure 3). In addition to these disynaptic inputs, arising from component fear network regions, reports also exist of direct, monosynaptic input to the cerebellum from VTA (Ikai et al., 1992), hypothalamus (Zhu et al., 2006) and locus coeruleus (Schwarz et al., 2015; Breton-Provencher et al., 2021).

## Cerebellar connections to the fear network in humans

In addition to the anatomical tracing studies described above in animal models, neuroimaging tractography studies provide an opportunity to study cerebellar anatomical connections to fear network regions in humans. Whilst these studies have confirmed in many instances that the cerebellum is indeed connected to a multitude of structures implicated in fear processing (see Habas, 2021 for recent review) in humans, they also highlight differences in cerebellar connectivity between human and non-human species. For example, Cacciola et al. (2019) recently performed constrained spherical deconvolution probabilistic tractography (a fibre orientation distribution modelling algorithm used for reliable tracking of fibre crossing regions) on high-angular resolution diffusion-weighted imaging (HARDI) data obtained from the Human Connectome Project repository to chart cerebellar connectivity in typically developing subjects. This analysis confirmed anatomical tracing studies in rodents, in that projections were identified from all cerebellar nuclei to the periaqueductal grey, with the strongest projection emanating from the fastigial nucleus. In addition, contrasting to our current understanding in rodents, the authors also revealed that regions of both cerebellar vermis (lobules IX, III, VI, X) and hemispheres (hemispheric lobule IX) are connected to the periaqueductal grey thus indicating the potential presence of a closed cerebello-midbrain anatomical loop in humans. Using a similar constrained deconvolution spherical modelling study in humans, Arrigo et al. (2014) identified direct connectivity between a multitude of cerebellar cortical regions (lobules VIII, IX, X, Crus I, Crus II), fastigial nucleus and the hippocampus. This study also contrasts to previous work in mice, in which only indirect anatomical connections have been described between restricted regions of the cerebellum and hippocampus (Watson et al., 2019).

Hemispheric Crus I/II has been consistently reported as a non-motor, functional hub in the cerebellum of humans (Van Overwalle et al., 2020a; Habas, 2021). Structural MRI studies have described, for example, that via output through the dentate nucleus, the cerebellar hemispheres may connect to thalamic relays that project onward to both striatal (Pelzer et al., 2013) and cortical (Palesi et al., 2021) regions potentially involved in fear-based emotional processes. Structural MRI studies have also described input to the cerebellum, routed via the pontine nuclei, from frontal, parietal and temporal cortical regions (Palesi et al., 2017; Karavasilis et al., 2019; Rousseau et al., 2022) and subthalamic nuclei (Pelzer et al., 2020; Wang et al., 2020). Functional MRI studies also demonstrate Crus I/II connectivity directed to components of the fear network, such as thalamus (Shen et al., 2020), prefrontal cortex (O'Reilly et al., 2010; Buckner et al., 2011; Chen et al., 2022), hippocampus (Fastenrath et al., 2022), amygdala (Fastenrath et al., 2022) and precuneus (Van Overwalle et al., 2020b). Additionally, Fastenrath et al. (2022) and Van Overwalle et al. (2020b) have described bi-directional cerebello-cingulate and cerebello-parietal interactions, respectively. Whilst the differences observed in cerebellar connectivity patterns with non-motor or fear-related brain regions between humans and lower species are currently not well understood, it has been suggested that they may to some extent reflect the massive co-expansion of the cerebellar hemispheres in primates in parallel to cognitive regions of the cortex (Balsters et al., 2010; Buckner et al., 2011) and intelligence (Barton and Venditti, 2014).

Overall, the pathways above describe the abundance of anatomical routes (further overviewed in Figure 4) via which the cerebellum may engage with the wider fear network. However, it is also critical to consider how the internal circuitry within the cerebellum may contribute to the computations related to emotion. Therefore, below we describe studies on the cellular and synaptic underpinnings of cerebellar contributions to fear.

## Intra cerebellar micro-circuit mechanisms supporting a role in fear

Cerebellar circuit involvement in fear memory consolidation was first described in rodent models by Sacchetti et al. (2002). By inactivating the cerebellar vermis or interpositus nuclei, using tetrodotoxin administered specifically during the fear consolidation phase, the authors revealed that functional integrity of these two cerebellar regions was required for appropriate formation of cued fear memories. Sacchetti et al. (2007) expanded upon this finding by showing that cerebellar inactivation within a one-hour window following fear recall was necessary to impair subsequent memory formation in rats. In addition, Sacchetti and colleagues also described long-term synaptic changes in the cerebellum during fear learning (Sacchetti et al., 2004). By measuring the level of potentiation at the parallel fibre—Purkinje cell synapse in slices obtained from unconditioned versus cue fear conditioned rats, the authors observed an α-amino-3-hydroxy-5-methyl-4-isoxazolepropionic acid (AMPA) dependent, postsynaptic long-term potentiation specifically in cerebellar cortical lobule V/VI. Further work from the same group revealed that this fear learning induced synaptic strengthening in the cerebellar cortex requires an intact basolateral amygdala (Zhu et al., 2011). These seminal findings (Sacchetti et al., 2002, 2004, 2007; Zhu et al., 2011) were key to inspiring subsequent research aimed at understanding the neural basis of cerebellar involvement in associative fear learning.

Indeed, the understanding of cellular processes supporting cerebellar contributions to fear memory formation has been improved by subsequent recent studies in a range of species. For example, in zebrafish, Takeuchi et al. (2017) and Koyama et al. (2021) have highlighted a key role of cerebellar granule cells in recovery from fear responses whilst Dubois and Liu (2021) revealed a strict requirement for decreasing inhibitory transmission in the cerebellar cortex molecular layer in order to reset cerebellar circuits for fear extinction learning in mice. Whilst Sacchetti et al. (2004) described that *hotfoot* mice deficient in parallel fibre—Purkinje cell synaptic potentiation had impaired fear response to conditioned stimuli (Sacchetti et al., 2004), Han et al. (2021) further revealed that signal transducer and activator of transcription 3 (STAT3)-dependent molecular regulation of glutamatergic input to Purkinje cells is indispensable for proper expression of fear memory. In contrast, GABAergic input to Purkinje cells mediated via molecular layer interneurons is not required for both fear memory acquisition and recall (Marshall-Phelps et al., 2020).

Together, these studies have made important steps in unpicking the cellular mechanisms within the cerebellar cortex that may subserve its role in fear processes and in particular fear memory formation. Another major challenge to the field is to understand if these findings generalise across the cerebellum or are restricted to anatomically, physiologically, or molecularly defined cerebellar subregions. It will also be important to understand if these mechanisms remain intact in animal models of disorders, such as autism. Indeed, in terms of fear memory and autism, animal model studies have provided evidence for both enhanced and decreased fear learning, linked to intrinsic changes in non-cerebellar fear-circuit regions (e.g., amygdala, Markram et al., 2008; Fernandes et al., 2021 and prefrontal cortex, Tatsukawa et al., 2019). It remains unknown whether similar cellular mechanisms also support cerebellar contributions to extinction learning and fear-based emotional processing in autism. Building upon the studies described above, in our subsequent section we will outline studies investigating the neural dynamics within extended cerebellar networks during fear to understand how the cerebellum interfaces with other brain regions during fear-based emotional processing.

## Physiological and functional substrates of distributed cerebellar network contributions to fear

Whilst accumulating evidence outlined above indicates both intra-cerebellar mechanisms relating to fear memory formation as well as robust neuroanatomical substrates allowing for cerebellar interaction with many brain regions previously linked to fear, there has also been a recent increase in the number of studies investigating the dynamics of distributed cerebellar network activity during fear-based emotional processing.

Although many studies have highlighted cerebellar functional interactions with a multitude of key brain regions considered part of the fear network, such as prefrontal cortex (Habas et al., 2009; Watson et al., 2009, 2014), hippocampus (Watson et al., 2019; Zeidler et al., 2020) and periaqueductal grey (Cerminara et al., 2009; Koutsikou et al., 2015) amongst others, only recently have such interactions been studied specifically during fear and fear-based learning.

Based upon classical Pavlovian conditioning paradigms in which a neutral stimulus is paired with an aversive stimulus, such as an electrical shock (Pavlov, 2010), ground-breaking studies in humans (Ernst et al., 2019; Batsikadze et al., 2022) and rodents (Frontera et al., 2020; Vaaga et al., 2020; Lawrenson et al., 2022) have begun to detail the neural dynamics within these circuits during fear. Importantly, Pavlovian conditioning allows assessment of fear memory formation, which occurs during or following the initial conditioning stage and fear extinction learning, when the conditioned stimulus is repeatedly presented alone. This extinction process, by which the fear response (often measured as cessation of all body movements or freezing) is reduced over time, has been linked by many studies to signalling of the discrepancy between predicted and actual sensory information (e.g., the presence or absence of an unexpected or expected footshock, termed PE; McNally et al., 2011). Indeed, PE signalling has been observed in different regions of the brain, such as the ventral striatum (VS; Thiele et al., 2021), prefrontal cortex (Casado-Roman et al., 2020), periaqueductal grey (Lawrenson et al., 2022), ventral tegmental area (Salinas-Hernandez et al., 2018; Cai et al., 2020) and cerebellum (Ernst et al., 2019). From electrophysiological or calcium imaging studies, putative PE signals recorded in, for example, VTA and PAG have been indicated by phasic increases in neuronal activation around the timing of the unexpected omission of an aversive stimuli (e.g., an electrical foot shock; see Salinas-Hernandez et al., 2018; Cai et al., 2020; Lawrenson et al., 2022 for examples of this). Similarly, PE related responses have been described in functional MRI studies as increases in blood-oxygen-level-dependent changes within the cerebellar cortex (Ernst et al., 2019). Strikingly, the magnitude of PE correlates with the level of fear recall exhibited (see Salinas-Hernandez et al., 2018; Ernst et al., 2019; Cai et al., 2020). Through the variety of monosynaptic connections that it has with fear-related brain areas (described in the preceding section of this review), the cerebellum is indeed well placed to take part in distributed PE computation. Our understanding of this process is still in its infancy but recent studies in both rodents and human have provided an important starting point in addressing this knowledge gap (Ernst et al., 2019; Frontera et al., 2020; Vaaga et al., 2020; Lawrenson et al., 2022). In particular, there has been a recent research focus on projections between the cerebellum and ventrolateral periaqueductal grey (vlPAG), which is an area of the midbrain heavily involved in fear behavioural response execution (e.g., freezing; Vaaga et al., 2020), fear memory formation (Yeh et al., 2021) and extinction learning (Watson et al., 2016; Arico et al., 2017). Working with mice, Frontera et al. (2020) charted excitatory projections from the cerebellar fastigial nucleus to vlPAG. Using a combination of virally guided chemo- and opto-genetics the authors were able to demonstrate that manipulation of this pathway can bidirectionally control the strength of fear memory formation in addition to extinction learning. These findings were further supported and extended by Lawrenson et al. (2022) who also focussed on the fastigial nuclei interactions with vlPAG in rats and found that reversible inactivation of this nuclei using the GABA agonist muscimol during fear memory consolidation led to reduction in temporal encoding accuracy of vlPAG cells during subsequent recall. Furthermore, the authors also adopted a chemogenetic approach to inhibit fastigial projections to vlPAG specifically during fear acquisition and observed a slower rate of fear extinction learning. Thus together, these two studies provide evidence that fastigial nucleus projections to the vlPAG are important in both the formation of fear memories and their extinction. Whilst Frontera et al. (2020) revealed that cerebellar projections target both inhibitory and excitatory cells within the vlPAG, Vaaga et al. (2020) identified a specific subpopulation of glutamatergic, chx10 expressing cells in the vlPAG, that drive freezing. Using optogenetic and *in vitro* experimental approaches, the authors then identified a cellular mechanism through which cerebellar projection neurons synapsing on to chx10 cells can modulate dopaminergic signalling in the vlPAG and bias postsynaptic currents towards inhibition. Thus, output from the medial cerebellum can regulate freezing via altering synaptic integration within the vlPAG microcircuit. Although most studies of cerebellar contributions to fear have focussed on the A module, composed of vermal regions and associated fastigial nuclei output, a recent study highlighted the importance of dopaminergic innervation of the lateral (dentate) nuclei for contextual fear learning (Carlson et al., 2021). Given that the lateral cerebellum is often considered to be involved in cognitive functions, especially in humans (Sokolov et al., 2017), further experiments investigating lateral cerebellar connections and interactions with fear networks are warranted.

Whilst these studies have provided important mechanistic insights into cerebellar contributions to fear processing in rodent models, *in vivo* imaging in humans using positron emission tomography (PET) or functional magnetic resonance imaging (fMRI) to detect changes in regional blood flow or oxygenation level, respectively, have identified cerebellar activity patterns across phases of Pavlovian conditioning paradigms (see Hwang et al., 2022 for recent overview). Ploghaus et al. (1999) were able to dissociate changes in blood oxygenation level dependent signals dependent upon expectation of aversive experience in the cerebellum alongside medial frontal lobe and insular cortex from those driven directly by aversive stimuli. Specifically, the authors showed that cerebellar activation during anticipation of an aversive stimulus was predominantly localised to the ipsilateral posterior cerebellum. Interestingly, this activation occurred only during the time immediately preceding aversive stimuli delivery and the anticipation signal increased in amplitude over conditioning trials. In contrast, pain-related signal amplitude remained consistent over time. Fischer et al. (2000) used PET to compare changes in regional cerebral blood flow (rCBF) in response to a stimuli paired with an aversive stimuli to an unpaired, conditioned stimuli alone. When comparing rCBF between the two conditions, significant increases were observed specifically in the left cerebellum thus further linking this region to Pavlovian conditioning.

Subsequent imaging studies have highlighted cerebellar activation as part of the threat processing network alongside amygdala (Linnman et al., 2011; Zhang et al., 2016; Ernst et al., 2019), as well as the frontal, parietal and temporal cortices (Han et al., 2008). In particular, cerebellar lobules V, VI, VIII (Zhang et al., 2016) and Crus I (Ernst et al., 2019) were significantly activated during expectation of aversive stimuli. In terms of functional coupling, Linnman et al. (2011) demonstrated decreased coherence between the cerebellar tonsil area and hippocampus in response to omitted aversive shocks. More recently, Batsikadze et al. (2022) tested whether memory persists within the cerebellum following extinction training in humans. Using a differential fear-learning paradigm to compare responses to conditioned stimuli paired with aversive stimuli and those driven by unpaired, conditioned stimuli alone, the authors found comparable cerebellar activations in Crus I/lobule VI during both associative and non-associative fear acquisition. Interestingly, during subsequent fear recall, these Crus I and lobule VI activation patterns were found to reoccur. In addition to cerebellar activation during aversive stimuli prediction, during subsequent extinction learning, significant blood oxygen level dependent activation of Crus I has been observed during unexpected omission of the conditioned stimuli (PE signalling) and this activation waned over extinction learning in a manner similar to that described in other brain regions (Ernst et al., 2019). This study is supported by findings from Faul et al. (2020) showing that fear extinction efficacy was related to the level of fear representation persistence in the cerebellum. In terms of PE encoding specifically, the studies of Ploghaus et al. (1999) and Ernst et al. (2019) highlight a role of cerebellar activation in both fear learning and extinction, respectively. However, the nature of the roles played by specific cerebellar regions in these processes remain far from fully understood.

## Distributed cerebellar circuits and autism

Thus far, this review has focussed on cerebellar circuit connectivity and function in typically developing animal models and humans. However, our aim is to explore the novel concept that the cerebellum, through its interactions with the wider brain, may be involved in the fear and anxiety issues observed in conditions associated with altered development of the nervous system, such as autism. Therefore, in this section of this review we will briefly outline research highlighting links between cerebellar dysfunction and autism. An important caveat of this discussion is that the majority of studies in humans examine autistic individuals without intellectual disability and focus on people with minimal support needs. Moreover, since many early studies failed to report scores of phenotypic severity, it can be difficult to know what aspects of autism any reported differences in brain structure and connectivity relate to. In contrast, the majority of animal studies model monogenic forms of autism in which intellectual disability, autism and often childhood epilepsy co-occur. Furthermore, rodents and humans have distinct evolutionary trajectories imposing different fitness pressures and hence distinct behavioural repertoires. Hence, whilst particular genetic alterations may lead to similar cellular and circuit alterations, the behavioural expression of these changes may be quite different. Therefore, making cross-species behavioural comparisons can be quite challenging and can be easily confounded by anthropomorphisation. Despite these limitations, cross-species comparisons are an excellent starting point for generating hypotheses regarding the circuit-basis of autistic features.

Indeed, the circuit pathophysiology underlying the cognitive and behavioural features associated with autism is currently poorly understood. Despite the phenotypic heterogeneity across affected individuals, the cerebellum has been consistently linked to the pathogenesis of autism (reviewed in Wang et al., 2014). Cerebellar nuclei and Purkinje cells have been found to be reduced in both number and size in post-mortem brains from autistic individuals (Bauman, 1991; Fatemi et al., 2002; Whitney et al., 2008). In addition, neuroimaging studies have reported cerebellar hypoplasia in lobules VI and VII of people with autism (Courchesne et al., 1988).

It is thus apparent that changes within cerebellar intrinsic circuitry may occur in autism. As described in the preceding sections, the cerebellum has abundant structural and functional connections to forebrain and midbrain regions associated with fear and emotional processing in neurotypical brains. Does distributed cerebellar connectivity and functional interaction with these regions change in autistic individuals? This question has received considerable attention in recent years, albeit with a focus on cognitive and social functions rather than fear-based emotional processing *per se*. In particular, a growing body of literature indicates aberrant cerebello-cortical interaction in autism, which we describe next (see Table 1; for reviews see Becker and Stoodley, 2013; D'mello and Stoodley, 2015; Crippa et al., 2016; Stoodley, 2016).

**Table 1:** Disrupted cerebello-cerebral connectivity reported in autism.

Cerebellar circuits	Hyperconnectivity	Hypoconnectivity
General cerebellar connections	Cerebellum – visual, somatosensory, motor network (human rsfMRI, Oldehinkel et al., 2019)	Cerebellum – fusiform/postcentral/temporal gyrus (human rsfMRI, Ramos et al., 2018)
	Right cerebellum – right cerebral cortex (human rsfMRI, Noonan et al., 2009; Khan et al., 2015)	Cerebellum – sensory cortices (human sMRI, Cardon et al., 2017)
	Posterior cerebellum – occipital, parietal cortex (human rsfMRI, Wang et al., 2019)	Cerebellum – visual cortices (human sMRI, Cardon et al., 2017)
	Right posterior cerebellum – bilateral dorsomedial M1 (human rsfMRI, Lidstone et al., 2021)	Lateral cerebellum – PFC (human rsfMRI, Wang et al., 2019)
	Cerebellum – PFC (human EEG during social interaction, Gaudfernau et al., 2022)	Right posterior cerebellum – left inferior parietal lobule (human rsfMRI, Lidstone et al., 2021)
Lobule-specific cerebellar connections	Right Crus I – left inferior parietal lobule (mice sMRI and human rsfMRI, Stoodley et al., 2017)	Right Crus I – left anterior intraparietal lobule (human visual motor task fMRI data, Unruh et al., 2023)
	Left Crus I – left insula (human rsfMRI, Hanaie et al., 2018)	Right Crus I – mPFC (mice sMRI and human rsfMRI, Kelly et al., 2020)
	Right Crus II – left supramarginal gyrus (human rsfMRI, Hanaie et al., 2018)	Right Crus I – frontal cortex, supplementary motor area, basal ganglia, thalamus (human rsfMRI, Okada et al., 2022)
	Left Crus II – Right middle frontal gyrus (human rsfMRI, Hanaie et al., 2018)	Right Crus I – contralateral superior frontal/middle frontal/ anterior cingulate gyrus, parietal areas, thalamus (human rsfMRI, Verly et al., 2014)
	Crus I, lobules VI, VII – STC, S1, PMC, OCC (premotor/M1/S1/the occipital lobe) (human rsfMRI, Khan et al., 2015)	Right Crus II – frontal/temporal/parietal areas (human rsfMRI, Olivito et al., 2018)
	Left lobule I-IV – ventral DMN (human rsfMRI, Bednarz and Kana, 2019)	Left Crus II – right dorsal temporoparietal junction (human rsfMRI, Igelström et al., 2017)
	Lobule IV/V – left middle frontal gyrus (human rsfMRI, Noonan et al., 2009)	Crus I/II, lobules VI, VII – contralateral PFC, posterior parietal cortex, inferior/middle temporal gyrus (human rsfMRI, Khan et al., 2015)
	Vermis VIIIa – right angular gyrus (human rsfMRI, Hanaie et al., 2018)	Crus I/II and lobule IX – parietal cortex, PFC (BA10), posterior cingulate, inferior frontal gyrus, insula (human rsfMRI, Arnold Anteraper et al., 2019)
	Vermis VIIIb – right supramarginal gyrus (human rsfMRI, Hanaie et al., 2018)	Vermis VI – left middle frontal gyrus (human rsfMRI, Hanaie et al., 2018)
	Vermis X – right middle temporal gyrus (human rsfMRI, Hanaie et al., 2018)	
DCN-specific connections		DN – supramarginal gyrus (human rsfMRI, Arnold Anteraper et al., 2019)
		Left DN – precentral, angular gyrus and parietal opercular cortex (human rsfMRI, Olivito et al., 2017)
		Right DN – left inferior frontal gyrus, left inferior parietal lobule (human rsfMRI, Hanaie et al., 2018)
		DN – thalamus (human sMRI, Jeong et al., 2012)

This table shows evidence for structural and functional hyper- and hypo-connectivity between cerebellum and cerebral cortical regions in autism. Studies in which details of specific cerebellar regions involved are included under the section on general cerebellar connections. Other sections include studies in which disruption in specific regions of the cerebellum is described. DCN, deep cerebellar nuclei; DMN, default mode network; DN, dentate nucleus; M1, primary motor cortex; OCC, occipital cortex; PFC, prefrontal cortex; PMC: premotor cortex, STC: superior temporal cortex, S1: primary somatosensory cortex. Data sources shown in parentheses, rsfMRI indicating resting state functional MRI data and sMRI representing structural MRI data.

From the human literature, in 2014, Stoodley conducted an important anatomical likelihood analysis and meta-analysis of voxel-based morphometry studies into autism, ADHD and dyslexia (Stoodley, 2014). In data obtained from people with autism, reduced grey matter was found in cerebellar vermal lobules IX, VIII and right Crus I. Importantly, these regions also showed functional connectivity clusters with fronto-parietal, default mode, somatomotor and limbic regions, and were distinct from those seen in ADHD or dyslexia. Resting state neuroimaging studies in humans, in which there is no explicit cognitive load, have revealed hypo-connectivity between cerebellar Crus I/II, lobule IX and a wide range of cortical regions in people with autism (Ramos et al., 2018; Arnold Anteraper et al., 2019). In contrast, a recent study combining EEG recordings from cerebral and cerebellar cortices during social tasks found an increase in connectivity, as evidenced by increased theta band coherence, between cerebellum and prefrontal cortex compared to neurotypical subjects (Gaudfernau et al., 2022).

**Figure 5:**
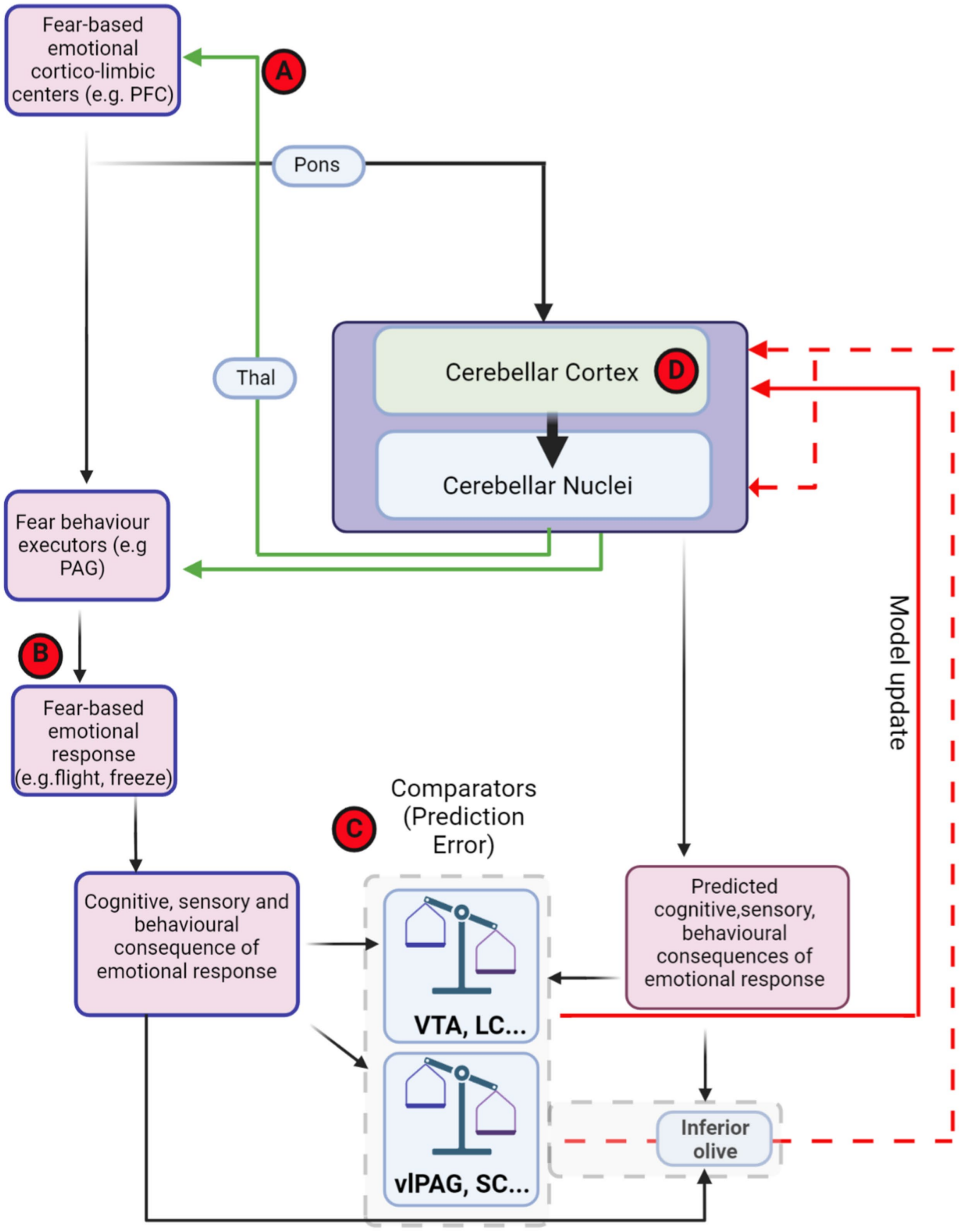
Theoretical framework of distributed, fear-related predictive cerebellar circuits and potential hubs for dysfunction in autism. Theoretical organisation of information processing flow that could used for generating and updating cerebellar models for fear-based emotional contributions. Red-circled letters indicate putative loci where circuit differences may occur in autism. Cognitive commands that are sent to executor structures, such as PAG, are also copied to the cerebellum (efference copy). The generated cerebellar internal models may be able to predict the consequences of PAG output. The site of comparison between actual consequences of defensive response, driven, e.g., by PAG activity, and those predicted by the cerebellum could in theory occur in multiple brain regions (examples, such as VTA, LC, and IO are shown inside dashed grey box). The output from these comparator regions may then relay back to update the cerebellum in the form of PE through either direct (solid red line) or indirect projections (dashed red lines). **(A)** Cerebello-thalamo-cortical interaction has been shown to be important in both fear extinction learning and in autism. **(B)** Abberant top-down control of fear behaviour executors may lead to abnormal fear responses in autism. **(C)** Changes in prediction error input signals to the cerebellum may drive inappropriate learning in cerebellar circuits. **(D)** Impairment within the cerebellar circuit itself, including changes in Purkinje cell number, anatomy and physiological properties may lead to deficits in internal model formation and stimulus–response associations. Thal, thalamus; VTA, ventral tegmental area; PAG, periaqueductal grey; vlPAG, ventrolateral periaqueductal grey; SC superior colliculus; LC, locus coeruleus; PFC, prefrontal cortex. Dashed red line indicates indirect olivo-cerebellar pathway. Figure made with Biorender.com.

The two studies described above and those shown in Table 1 have indicated state-dependent aberrance in cerebro-cerebellar coupling in autistic individuals. In addition, recent work has attempted to identify the effect of selective disruption on autism-linked genes in the cerebellum on its functional coupling with other brain regions. Of particular note, Stoodley et al. (2017) investigated and compared cerebro-cerebellar interactions in both autistic children and a mouse model lacking the tuberous sclerosis complex 1 gene specifically in Purkinje cells (tuberous sclerosis is a rare genetic condition caused by mutations in the *TSC1* or *TSC2* genes; an estimated 40%–50% of affected children are diagnosed with autism and/or intellectual disability; see Tsai et al., 2012). Neuromodulation of Crus I resulted in altered functional connectivity with the parietal lobe in neurotypical humans and this network was found to be atypically connected in autistic children. Interestingly, Crus I—parietal cortex structural connectivity was altered in mice lacking *Tsc1* in Purkinje neurons. Using a chemogenetic approach, the authors demonstrated that inhibition of Crus I alone was sufficient to generate abnormal social interactions and repetitive behaviours in control mice, whereas stimulation of Crus I Purkinje cells could rescue social impairments in the *Tsc1* mouse model. These findings align with work of Badura et al. (2018) who showed that chemogenetic modulation of cerebellar cortex (including Crus I) during juvenile stages of development correlates with perseverative behaviour and reduces social preference in adult mice, potentially via changes in long-range connectivity to non-motor, cerebral cortical regions such as the prelimbic, cingulate and orbitofrontal cortices, respectively. The importance of cerebellar interactions with prefrontal cortex were further highlighted by Kelly et al. (2020) who demonstrated altered structural covariance between cerebellum and prefrontal cortex in 30 different genetic mouse models of autism. Furthermore, the authors demonstrated that chemogenetic manipulation of components of this circuit could improve autism-related behaviours observed in the Purkinje cell specific *Tsc1* mouse model. Thus, the balance of evidence indicates robust changes in cerebellar interactions with cognitive regions, such as the prefrontal cortex, in autism. To date, studies have not yet focussed on the effect of aberrant prefrontal-cerebellar interaction in relation to fear-related process in autism. However, given that encoding of prediction error in prefrontal cortex has been linked to fear learning in humans (Seymour et al., 2004, 2005; Jensen et al., 2007) and sustained activity within the prelimbic cortex of rodents is closely correlated with fear extinction failure (Burgos-Robles et al., 2009), changes in the neural dynamics within this region could have consequences for cerebellar encoding of internal models (see Figure 5). For example, prefrontal cortex top-down control of defensive behaviours involves interactions with downstream effectors, such as the PAG (Rozeske et al., 2018). Commands sent to these structures may also reach the cerebellum in the form of efference copy, routed through the pontine nuclei (Schmahmann and Pandya, 1995; Suzuki et al., 2012). Abnormal activity within this pathway may drive inappropriate internal model formation within the cerebellum, which in turn could contribute to fear extinction failure observed in rodent models of autism (Katsanevaki et al., 2020). Recent work has also causally linked 4 Hz oscillatory activity within the prefrontal cortex of rodents in the maintenance of defensive freezing responses to fear-related stimuli (Karalis et al., 2016). Intriguingly, this activity pattern is reliant upon input from the cerebellum, routed through the medio-dorsal thalamus (Frontera et al., 2023). Thus, disruption of cerebellar output could potentially lead to fear response imbalance (as recently observed in Katsanevaki et al., 2020; Anstey et al., 2022) via modulation of 4 Hz oscillations in the prefrontal cortex.

The studies described above indicate that cerebellar interactions with a multitude of brain regions, including those not classically considered involved in motor control, are important in the aetiology of autism (and other neurodevelopmental disorders). In particular, the recent studies described above have consistently implicated changes in these connections to the development of social impairments and presence of repetitive, inflexible behaviours. The fundamental question of how the cerebellum contributes to these behavioural changes remains far from understood. However, as mentioned throughout this review, the known predictive encoding or internal model function of the cerebellum provides an attractive angle through which to potentially understand its contribution to non-motor functions. For example, appropriate social interactions (including those with fear-based emotional components) require dynamic, constantly updated predictions of the consequences of given actions on others (Balsters et al., 2017; Kinard et al., 2020). The generation of appropriate internal models to smoothly cope with such emotionally-loaded, context dependent situations may necessitate cerebellar connectivity and interaction with cortico-limbic regions and indeed these models may be particularly susceptible to miscalibration throughout development (though this remains to be tested; see Kelly et al., 2021; Stoodley and Tsai, 2021 for recent discussion on the topic).

In the final section of this review, we will discuss the proposal that the cerebellum may be involved in fear-based emotional dysregulation in conditions associated with altered development of the nervous system.

## Linking fear-based emotional processing, cerebellar circuit function and neurodevelopmental disorders, such as autism

The fact that predictive encoding is vital for learning and inability to predict outcomes is known to affect adaptive behaviour has led to the hypothesis that predictive impairments are central to the pathophysiology underlying autism traits (Pellicano and Burr, 2012; Sinha et al., 2014; Van de Cruys et al., 2014). Environmental unpredictability is a trigger leading to anxiety (Chorpita and Barlow, 1998) and clinically, anxiety disorders and phobias are prevalent in autistic individuals and often used to inform diagnosis (Kerns and Kendall, 2014). Moreover, since predictability is also key for the process of habituation, a change in processing of predictability could lead to differences in sensory perceptions (e.g., hypo- or hyper-sensitivity). Consistent with this, many people with autism show abnormal sensory reactivity (Burke and Cerniglia, 1990; Green et al., 2013) and some express abnormal fear responses to external stimuli (Dawson et al., 2004; Gaigg and Bowler, 2007; Top et al., 2016; Macari et al., 2018; Sapey-Triomphe et al., 2021). Studies in preclinical models of autism with intellectual disability have reported changes in fear behaviour as well as cerebellar circuits (Table 2). Surprisingly, links between the cerebellum and fear related deficits observed in autism are not widely investigated.

**Table 2:** Fear-related behaviour and circuit abnormalities in preclinical models of autism.

**Animal model**	**Fear behaviour**	**Fear circuit/cerebellar deficits**
VPA	Dose dependent memory acquisition and extinction deficit (Banerjee et al., 2014).	Increased lobule VI volume in males. Decreased lobule I, VI and X volume in females (Payne et al., 2021).
	Enhanced fear memory (Markram et al., 2008).	
Homer1	Impaired short- and long-term fear memory (Banerjee et al., 2016)	Homer1 overexpression in BLA (Banerjee et al., 2016)
ARID1B	Successful fear memory acquisition and normal fear recall (Ellegood et al., 2021)	Smaller cerebellum and larger hippocampus (Ellegood et al., 2021)
PCDH10	Reduced freezing levels (Ferri et al., 2021)	Decreased gamma oscillation in BLA (Ferri et al., 2021)
NLGN3	Aberrant fear behavioural expression. Preference towards flight responses (Anstey et al., 2022)	Hyperexcitability in dorsal PAG (Anstey et al., 2022)
SYNGAP1	Impaired fear extinction (Katsanevaki et al., 2020)	Decreased cortico-cortico coherence during non-rapid eye movement sleep (Buller-Peralta et al., 2022)
FMR1	Impaired fear recall (Fernandes et al., 2021)	Amygdala dysfunction (Fernandes et al., 2021). Impaired Purkinje cell long term depression and startle response (Koekkoek et al., 2005).
EN2	Less freezing in contextual and cued fear conditioning (Brielmaier et al., 2012)	Impaired cerebellar development (James et al., 2013)
CDH13	Impaired fear extinction (Kiser et al., 2019)	Decreased cerebellar connectivity with dorsal raphe nuclei during development (Kiser et al., 2019)
SCN2A	Enhanced fear conditioning and deficient fear extinction (Tatsukawa et al., 2019)	Increased gamma band neuronal activity in mPFC (Tatsukawa et al., 2019)

The above table highlights potential links between fear-based phenotypes in autism models and fear circuitry, including cerebellum where data is available. ARID1B, AT-Rich Interaction Domain 1B; BLA, basolateral amygdala; CDH13, Cadherin-1; EN2, Engrailed Homeobox 2; FMR1, Fragile X Messenger Ribonucleoprotein 1; HOMER1, homer scaffold protein 1; mPFC, medial prefrontal cortex; NLGN3, Neuroligin-3; PAG, periaqueductal grey; PCDH10, Protocadherin10; SCN2A, sodium voltage-gated channel alpha subunit 2; SYNGAP1, Synaptic Ras-GTPase-activating protein; VPA, valproic acid.

As outlined in the previous sections of this review, abnormalities in the cerebellum, and its connections to multiple regions of the cerebral cortex, are observed in people with autism. Some of the areas in which aberrant cerebellar connectivity has been noted in people with autism and preclinical models are particularly pertinent to fear learning, such as the prefrontal cortex (Becker and Stoodley, 2013; Kelly et al., 2021). Whilst cerebellar interactions with many of the connected fear-related regions outlined in Figures 2–4 are yet to be studied in autism models, disruption at any point within this distributed network (either at input or output stages) could potentially lead to changes in cerebellar fear-related information processing. Indeed, in Table 2, we highlight literature in which both fear-related behavioural and cerebellar or fear-circuit deficits have been described in animal models of autism.

Based upon current findings, in Figure 5 we present potential hubs for dysfunction within the distributed fear network and overlapping autism-related networks that may relate to impaired cerebellar predictive coding, and could theoretically lead to generation of aberrant fear responses in this or other disorders, such as post-traumatic stress disorder (Blithikioti et al., 2022) or schizophrenia (Bernard and Mittal, 2015). As described in the preceding section of this review, disruption of cerebello-cortical networks has been reported in both humans with autism and mouse models (e.g., Stoodley et al., 2017; Kelly et al., 2021) whereas Frontera et al. (2023) showed that modulation of similar pathways in wild-type mice can drive changes in fear extinction learning. Thus, cerebello-thalamo-cortical disruption in autism may contribute to imbalance in the generation of predictive signals in the extended cerebellar-fear network that are required for appropriate extinction learning (Figure 5A). Fear-behaviour execution centres, such as the PAG are also crucial for appropriate fear-response performance (Tovote et al., 2015). Recently, Anstey et al. (2022) demonstrated hyperactivity within dorsal PAG and an imbalance of fear-related defensive responses in a rat model of autism. Such an imbalance in fear-related behavioural response may in turn provide unexpected feedback to prediction error systems, leading to cerebellar internal model maladaptation. Furthermore, sensory hypersensitivity described in people with autism, could result in high magnitude reafferent signals driving inappropriate PE and aberrant updating of cerebellar internal models, which in turn could facilitate impaired learning (Figure 5B). In addition, changes in brain regions implicated in broadcasting PE signals, such as the ventral tegmental area and locus coeruleus have been described in animal models and people with autism (Bariselli et al., 2018; London, 2018; Huang et al., 2021; Keehn et al., 2021). Changes at this level could again lead to inaccurate updating of cerebellar circuits via projections to the cerebellar cortex (Figure 5C). Another candidate locus for impairment leading to PE is within the cerebellar circuitry itself (Figure 5D). As described earlier in this review, changes in Purkinje cell number and anatomy have been reported in post-mortem analysis of brains from people with autism (Fatemi et al., 2012). Purkinje cells are critical components of the cerebellar internal model system (Popa et al., 2013, 2016; Streng et al., 2018) and abnormalities in their anatomical or intrinsic properties could lead to inappropriate responses to error signals from the inferior olive or PE signals broadcast from neuromodulatory hubs, such as the ventral tegmental area and locus coeruleus. In line with this, plasticity at parallel fibre to Purkinje cell synapses has been shown to be altered in models of autism (Simmons et al., 2022) and could, for example, lead to inability to form correct memory associations and effect fear learning. Aberrance in this key cerebellar cortical computational unit may lead to inappropriate updating of cerebellar internal models resulting in excessive or maladaptive behavioural responses to stimuli. Purkinje cell disruption may also lead to knock on effects in connected deep cerebellar nuclei and impact extra-cerebellar targets, potentially further exacerbating predictive encoding issues. For example, as Frontera et al. (2020) demonstrated that fastigial nucleus-vlPAG interaction is essential for appropriate fear memory update, abnormality in Purkinje cell populations projecting to fastigial nucleus, may result in disturbed fear-related processes, abnormal stimulus–response association formation and extinction learning in autism.

## Future directions

Our survey of the literature clearly indicates substantial theoretical and functional links exist between cerebellum and both fear and autism. However, studies spanning these domains are clearly lacking. What is more, whilst recent studies have made great strides in our understanding of cerebellar contributions to fear-based emotional processing, the field is still at an early stage and many gaps in knowledge remain. Below we give a summary of what we consider the most pertinent research questions and areas yet to be addressed in the field:

### Understanding cerebellar interactions with fear networks

1. It is clear that a great degree of both molecularly-defined and projection-defined heterogeneity exists within both the cerebellar cortex (Cerminara et al., 2015) and nuclei (Fujita et al., 2020); thus, studies are required to fully understand the contribution that specific cerebellar neuronal subpopulations make to fear-based emotional processes.
2. Equally, many studies on cerebellar functional connectivity with the fear network to date have focussed on the medial cerebellum-to-vlPAG pathway; however, it is clear from anatomical studies that lateral cerebellar cortical regions and associated lateral cerebellar nuclei are monosynaptically connected with a range of other brain regions involved in fear-based emotional processing, such as ventral tegmental area, superior colliculus and mediodorsal thalamus. It is also unclear to what extent subpopulations within the cerebellar nuclei provide input to discrete or overlapping midbrain structures. The functional significance of these connections requires further investigation.
3. Whole brain imaging approaches, such as MRI, will allow identification of cerebellar interactions with other brain regions in an unbiased fashion during fear behaviour. Translational potential could be increased by combining MRI imaging of cerebellar circuits and, where possible, similar Pavlovian fear conditioning protocols in both rodents and humans.
4. Understanding the computations performed by functional/dysfunctional cerebellar circuits and how these embed within whole-brain fear networks will require computational modelling approaches.
5. It is unclear if mechanisms subserving cerebellar roles in fear processing also relate to, for example, reward processing. Does prediction error encoding in populations of cerebellar neurons support associative learning irrespective of emotional valence (e.g., reward versus punishment omission)?

### Understanding cerebellar contributions to anxiety and fear-related disorders in people with autism

1. Although a number of studies have identified cerebellar differences in people with autism, currently very little is known about how the cerebellum contributes specifically to commonly observed fear and anxiety issues. Several studies in animal models of neurodevelopmental disorders have examined the cellular, physiological and behavioural effects of disrupting genes linked to autism specifically in the cerebellum (Koekkoek et al., 2005; Tsai et al., 2012; Peter et al., 2016; Tsai, 2016; Stoodley et al., 2017; Kelly et al., 2020). Whilst this is a powerful approach to improve our understanding of cerebellar circuits, these models fail to recapitulate the condition accurately in that genetic mutations observed in autism are not limited to the cerebellum—most autism associated risk genes are expressed across many regions of the central (and peripheral) nervous system. Experiments examining distributed cerebellar function during fear behaviour in constitutive transgenic animal models of neurodevelopmental disorders are required to bridge this gap.
2. Finally, as highlighted within this review, the cerebellum is involved in predictive encoding via internal model generation and studies suggest this encoding may be aberrant in neurodevelopmental disorders, such as autism (Kelly et al., 2021). An important question will be to establish the developmental trajectory of changes underlying PE miscalibration exhibited by preclinical models (Dooley et al., 2021) and if it is possible to make early life, circuit-specific, interventions to reverse or prevent the emergence of behavioural differences driven by them, such as those related to fear.

## Conclusion

We hope that this review will drive further interest in understanding the role of the cerebellum in fear-based emotional processing. In particular, by highlighting the potential for cerebellar involvement in the fear-based emotional dysregulation often observed in autistic individuals, we aim to inspire future studies, at both the animal model and human subject level.

## Author contributions

All authors listed have made a substantial, direct, and intellectual contribution to the work and approved it for publication.
