# Supplementary material for: Cerebellar contributions to fear-based emotional processing: relevance to understanding the neural circuits involved in autism
Source: Front Syst Neurosci. 2023 Nov 21;17:1229627. doi: 10.3389/fnsys.2023.1229627 (PMC10703189; doi:10.3389/fnsys.2023.1229627)
Supplement: Supplementary file 1 [file Table_1.docx]

**Supplementary table of source literature used to construct Figure 2.**

| Reference | Cerebellar nuclei | Projection Target | Animal Model Species |
| --- | --- | --- | --- |
| Fujita et al. (2020) | FN | LC,VTA, SC, PAG, ZI, MD | Mouse |
| Kebschull et al. (2020) | FN, Int, DN | LC,R, ZI | Mouse, chicken, human |
| Schwarz et al. (2015) | Not specific | LC | Mouse |
| Mezey et al. (1985) | Int | LC, R | Cat |
| Clavier (1979) | Not specific | LC | Rat |
| Cedarbaum and Aghajanian (1978) | FN | LC | Rat |
| Judd et al. (2021) | Int | VTA, SC, PAG,R, MD | Mouse |
| Carta et al. (2019) | Not specific | VTA | Mouse |
| Perciavalle et al. (1989) | FN, Int | VTA | Rat |
| Baek et al. (2022) | DN | VTA | Mouse |
| Heiney et al. (2021) | Int | SC | Mouse |
| Gonzalo-Ruiz et al. (1988) | FN, Int | SC | Monkey |
| Gonzalo-Ruiz and Leichnetz (1987) | FN, Int, DN | SC | Rat |
| Katoh et al. (2000) | FN | SC | Cat |
| Hirai et al. (1982) | FN, Int, DN | SC | Cat |
| Beitz (1989) | FN, Int, DN | PAG | Rat |
| Beitz (1982) | FN, Int, DN | PAG | Rat |
| Frontera et al. (2020) | FN | vlPAG, MD | Mouse |
| Vaaga et al. (2020) | FN | vlPAG | Mouse |
| Gonzalo-Ruiz and Leichnetz (1990) | Int | dlPAG,R, ZI, MD | Monkey |
| Kawamura et al. (1982) | DN | dmPAG | Cat |
| Gonzalo-Ruiz et al. (1990) | FN | lPAG | Rat |
| Asanuma et al. (1983) | FN | R | Monkey |
| Marcinkiewicz et al. (1989) | Int,DN | R | Rat |
| Langer and Kaneko (1984) | FN | R | Cat |
| Haines and Dietrichs (1984) | FN, Int, DN | PH, LH | Monkey |
| Cavdar et al. (2001) | FN, Int, DN | DMH | Rat |
| Li et al. (2017) | FN | VMH | Rat |
| Haines et al. (1990) | FN, Int, DN | PH, LH | Tree shrew |
| Kuramoto et al. (2011) | Not specific | ZI | Rat |
| Sugimoto et al. (1981) | FN, Int | ZI | Cat |
| Aumann and Horne (1996) | DN | ZI | Rat |
| Roger and Cadusseau (1985) | Int, DN | ZI | Rat |
| Mitrofanis and deFonseka (2001) | Int | ZI | Rat |
| Haroian et al. (1981) | FN, Int, DN | TH | Rat |
| Person et al. (1986) | FN | MD | Dog |
| Stepniewska and Kosmal (1986) | FN, Int, DN | MD | Dog |
| Sakai and Patton (1993) | FN, Int, DN | MD | Dog |
| Sakai et al. (1996) | Int, DN | MD | Monkey |

|  |  |  |
| --- | --- | --- |

Abbreviations: FN, fastigial nucleus; Int, interpositus nucleus; DN, dentate nucelus; LC, locus coeruleus; VTA, ventral tegmental area; SC, superior colliculus; PAG, periaqueductal grey; dmPAG, dorsomedial periaqueductal grey; lPAG, lateral periaqueductal grey; dlPAG, dorsolateral periaqueductal grey; vlPAG, ventrolateral periaqueductal grey; ZI, zona incerta; MD, mediodorsal nucleus; R, raphe nuclei; PH, posterior hypothalamus; LH, lateral hypothalamus; DMH, dorsomedial hypothalamus; VMH, ventromedial hypothalamus; TH, thalamus.
